# Optimising COVID-19 episode identification using serology and PCR/rapid antigen testing: insights from the BRACE trial

**DOI:** 10.1186/s12879-026-13128-6

**Published:** 2026-04-07

**Authors:** Ellie McDonald, Laure F. Pittet, Marc Bonten, Anthony Byrne, John Campbell, Julio Croda, Margareth Dalcolmo, A. J. Davidson, Glauce dos Santos, Kaya Gardiner, Amanda Gwee, Bruno Jardim, Marcus Lacerda, Michaela Lucas, David J. Lynn, Laurens Manning, Helen Marshall, Kirsten P. Perrett, Cristina Prat-Aymerich, Marco Antonio Moreira Puga, Peter Richmond, Jesús Rodríguez-Baño, Ushma Wadia, Adilia Warris, Nicholas Wood, Nigel Curtis, Nicole L. Messina, the BRACE Trial Consortium Group

**Affiliations:** 1 Infectious Diseases Group, Murdoch Children’s Research Institute, Royal Children’s Hospital, 50 Flemington Rd, Parkville, VIC 3052 Australia; 2 Immunology, Vaccinology, Rheumatology and Infectious Diseases Unit, Faculty of Medicine, Geneva University Hospitals, Geneva, Switzerland; 3 Department of Paediatrics, The University of Melbourne, Parkville, VIC Australia; 4 European Clinical Research Alliance on Infectious Diseases, Utrecht, The Netherlands; 5 Julius Center for Health Sciences and Primary Care, University Medical Centre Utrecht, Utrecht University, Utrecht, The Netherlands; 6 St Vincent’s Hospital, Heart Lung Stream, Darlinghurst, Australia; 7 Faculty of Medicine, The University of New South Wales, St Vincent’s Clinical School, Sydney, Sydney Australia; 8 Exeter Collaboration for Academic Primary Care, University of Exeter Medical School, Exeter, UK; 9 Department of Epidemiology of Microbial Diseases, Yale School of Public Health, New Haven, CT USA; 10 Fiocruz Mato Grosso do Sul, Fundação Oswaldo Cruz, Campo Grande, Brazil; 11 Universidade Federal de Mato Grosso do Sul, Campo Grande, Brazil; 12 Centro de Referência Professor Hélio Fraga, ENSP/FIOCRUZ (Fundação Oswaldo Cruz), Rio de Janeiro, Brazil; 13 Melbourne Children’s Trial Centre, Murdoch Children’s Research Institute, Parkville, VIC Australia; 14 Research Operations, The Royal Children’s Hospital Melbourne, Parkville, VIC Australia; 15 Infectious Diseases, Royal Children’s Hospital Melbourne, Parkville, VIC Australia; 16 Antimicrobials Group, Murdoch Children’s Research Institute, Parkville, VIC Australia; 17 Institute of Clinical Research Carlos Borborema, Doctor Heitor Vieira Dourado Tropical Medicine Foundation, Manaus, Brazil; 18 Fundação de Medicina Tropical Doutor Heitor Vieira Dourado, Manaus, Brazil; 19 Instituto Leônidas & Maria Deane, Oswaldo Cruz Foundation, Ministry of Health, Manaus, Brazil; 20 University of Texas Medical Branch, Galveston, TX USA; 21 Department of Immunology, Queen Elizabeth II Medical Centre, Pathwest, Nedlands, WA Australia; 22 Department of Immunology, Perth Children’s Hospital, Nedlands, WA Australia; 23 Department of Immunology, Sir Charles Gairdner Hospital, Nedlands, WA Australia; 24 School of Medicine, University of Western Australia, Perth, WA Australia; 25 Flinders Health and Medical Research Institute, Flinders University, Bedford Park, South Australia Australia; 26 Precision Medicine Theme, South Australian Health and Medical Research Institute, Adelaide, South Australia Australia; 27 Department of Infectious Diseases, Fiona Stanley Hospital, Murdoch, WA Australia; 28 Wesfarmers Centre for Vaccines and Infectious Diseases, The Kids Research Institute, Nedlands, WA Australia; 29 Adelaide Medical School and Robinson Research Institute, The University of Adelaide and the Women’s and Children’s Research Centre, Women’s and Children’s Health Network, Adelaide, SA Australia; 30 Department of Allergy and Immunology, Royal Children’s Hospital Melbourne, Parkville, VIC Australia; 31 Population Allergy Group, Murdoch Children’s Research Institute, Parkville, VIC Australia; 32 Department of Immunology and General Paediatrics, Perth Children’s Hospital, Nedlands, WA Australia; 33 Division of Infectious Diseases and Microbiology, Department of Medicine, Hospital Universitario Virgen Macarena, University of Seville, Biomedicines Institute of Seville-Consejo Superior de Investigaciones Científicas, Seville, Spain; 34 CIBER de Enfermedades Infecciosas, Instituto de Salud Carloss III, Madrid, Spain; 35 Department of Infectious Diseases, Great Ormond Street Hospital, London, UK; 36 Medical Research Council Centre for Medical Mycology, Department of Biosciences, Faculty of Health and Life Sciences, University of Exeter, Exeter, UK; 37 Faculty of Medicine and Health, University of Sydney, Sydney, NSW Australia; 38 National Centre for Immunisation Research and Surveillance of Vaccine Preventable Disease, Westmead, NSW Australia; 39 Sydney Children’s Hospital Network, Westmead, NSW Australia

**Keywords:** Algorithm, Case definition, COVID-19, PCR, Rapid antigen test, Lateral flow test, Sankey

## Abstract

**Background:**

Accurately identifying COVID-19 episodes was crucial during the pandemic for evaluating interventions. Results from diagnostic tools like PCR, rapid antigen test (RAT) and serology are affected by factors such as timing of tests and vaccination status. The BRACE trial developed an algorithm integrating these diagnostic tools for illness episode classification.

**Methods:**

In the BRACE trial, 3988 participants reported 5512 febrile/respiratory illness episodes and provided longitudinal blood samples over one year. SARS-CoV-2 diagnosis relied on a three-component algorithm: (1) a serology algorithm assessing anti-SARS-CoV-2 nucleocapsid antibody seroconversion, (2) a PCR/RAT algorithm, and (3) an episode interpretation algorithm combining serology and PCR/RAT results to categorise episodes as COVID-19, Not COVID-19 or Uncertain. The algorithms accounted for vaccination status and timing of testing relative to symptom onset to refine episode classifications.

**Results:**

Of 5512 illness episodes, 890 (16%) were classified as COVID-19, 3852 (70%) as Not COVID-19, and 770 (14%) as Uncertain. Compared to relying solely on PCR/RAT results, integrating serology in the algorithm reduced the proportion of Uncertain classifications by more than half. Among the COVID-19 episodes, 89% were identified by positive PCR/RAT results, and the remaining 11% (with missing or negative PCR/RAT tests) were identified by serology. Discordance between PCR/RAT and serology occurred in 9% of episodes.

**Conclusions:**

An algorithm integrating PCR/RAT and serology results in the context of test timing and vaccine status enabled the accurate identification of COVID-19 episodes and minimised the number of episodes that would otherwise have been classified as Uncertain.

**Trial registration:**

The BRACE trial: BCG vaccination to reduce the impact of COVID-19 in healthcare workers. ClinicalTrials.gov NCT04327206, Registration date:27 March 2020.

**Supplementary Information:**

The online version contains supplementary material available at 10.1186/s12879-026-13128-6.

## Background

Accurately identifying COVID-19 episodes was vital for evaluating interventions in the pandemic. The performance of diagnostic tests for SARS-CoV-2, such as respiratory swab testing and serology, is influenced by the timing of testing relative to symptom onset, as well as prior COVID-19 vaccinations.

In the BRACE trial, comprehensive symptom data were collected alongside SARS-CoV-2 testing [1–3]. Here, we describe the algorithm developed during the BRACE trial, intended to more precisely identify COVID-19 episodes using illness episode data and diagnostic test results.

To account for test timing in relation to symptom onset and any effect of COVID-19 vaccination, a three-component interpretation algorithm was developed. This incorporated serological result interpretation, PCR/rapid antigen test (RAT) respiratory swab test interpretation, and finally episode classification to COVID-19, Not COVID-19 or Uncertain categories.

In addition to assessing the discordance/concordance of PCR/RAT testing to serology for SARS-CoV-2, we assessed the impact of each component of the algorithm on episode classification.

## Methods

### Participants and study design

The BRACE trial (NCT04327206) was a phase 3 international randomised controlled trial to assess the impact of BCG vaccination on the prevalence of COVID-19 among healthcare workers [1–3]. Trial outcomes relied on accurate ascertainment of the onset of a participant’s first episode of COVID-19.

The trial was designed early in the pandemic, with recruitment starting in March 2020. This study included the 3988 participants recruited to the BRACE trial from May 2020 to April 2021 from Australia, the Netherlands, Spain, the UK and Brazil. Selection of COVID-19 symptoms to be collected and diagnostic tests for SARS-CoV-2 were informed by best practice and government policies at the time.

### Illness episode ascertainment

Recent illness symptoms were ascertained weekly via a custom-built smartphone app or direct communication (phone call or text). During illness episodes, symptoms were recorded daily, and participants were prompted to undergo SARS-CoV-2 respiratory swab testing using PCR (via government testing centres) or RAT (self-administered). Active daily follow-up of participants began with report of any of 11 symptoms: fever, intermittent cough, persistent cough, shortness of breath, sore throat, runny nose, headache, fatigue, loss of taste and/or smell, muscle ache, vomiting, and/or diarrhoea. Follow-up continued until participants reported resolution of the illness. Where resolution was reported and symptoms recurred within 3 days the episodes were bridged [2, 4]. Additionally, more comprehensive questionnaires were administered at the start of the trial and quarterly throughout follow-up [1]. These collected information about COVID-19-specific vaccinations (date and vaccine type). Blood samples were collected at baseline and 3, 6, 9 and 12 months post-randomisation to measure anti-SARS-CoV-2 nucleocapsid (NCP) antibodies using the Roche Cobas Elecsys anti-SARS-CoV-2 assay [5, 6] at the Victorian Infectious Diseases Reference Laboratory. For seroconversion, a cut-off index (COI) of ≥ 1.0 was used as per manufacturer’s instructions [7]. 

### Serological interpretation algorithm

The serology algorithm produced one of four possible outcomes: Seroconversion, No seroconversion, Indeterminate or No result (where a blood sample was unavailable). For each illness episode, the most recent blood sample collected at least seven days prior to symptom onset (pre-episode sample), and the first and second blood samples collected after symptom onset were identified. The NCP serology result from these samples were used in the serological interpretation algorithm. The algorithm considered the timing of blood sample in relation to the illness episode, any prior administration of the *CoronaVac* (Sinovac Biotech) COVID-19 vaccine (a whole inactivated virus vaccine which can induce anti-NCP antibodies) and change in anti-NCP antibody titre (in cases of positive-to-positive serology results). A detailed description of the serological algorithm is provided in Supplementary Fig. 1.

### PCR/RAT interpretation algorithm

The PCR/RAT interpretation algorithm yielded three possible outcomes: Positive, Negative, or No result. Both PCR and RAT positive results were considered confirmation of SARS-CoV-2 infection if the timing of the test fell within defined symptomatic illness parameters. Positive PCR results were considered valid if the sample was tested within three days prior to 10 days (RAT) or 21 days (PCR) after the start, or within seven days from the end, of an illness episode. A negative PCR was considered ‘Negative’, but a negative RAT was classified as ‘No result’ due to the lower sensitivity of RAT testing [8, 9]. A detailed description of the PCR/RAT interpretation algorithm is provided in Supplementary Fig. 2.

### Episode interpretation algorithm

**Fig. 1:**
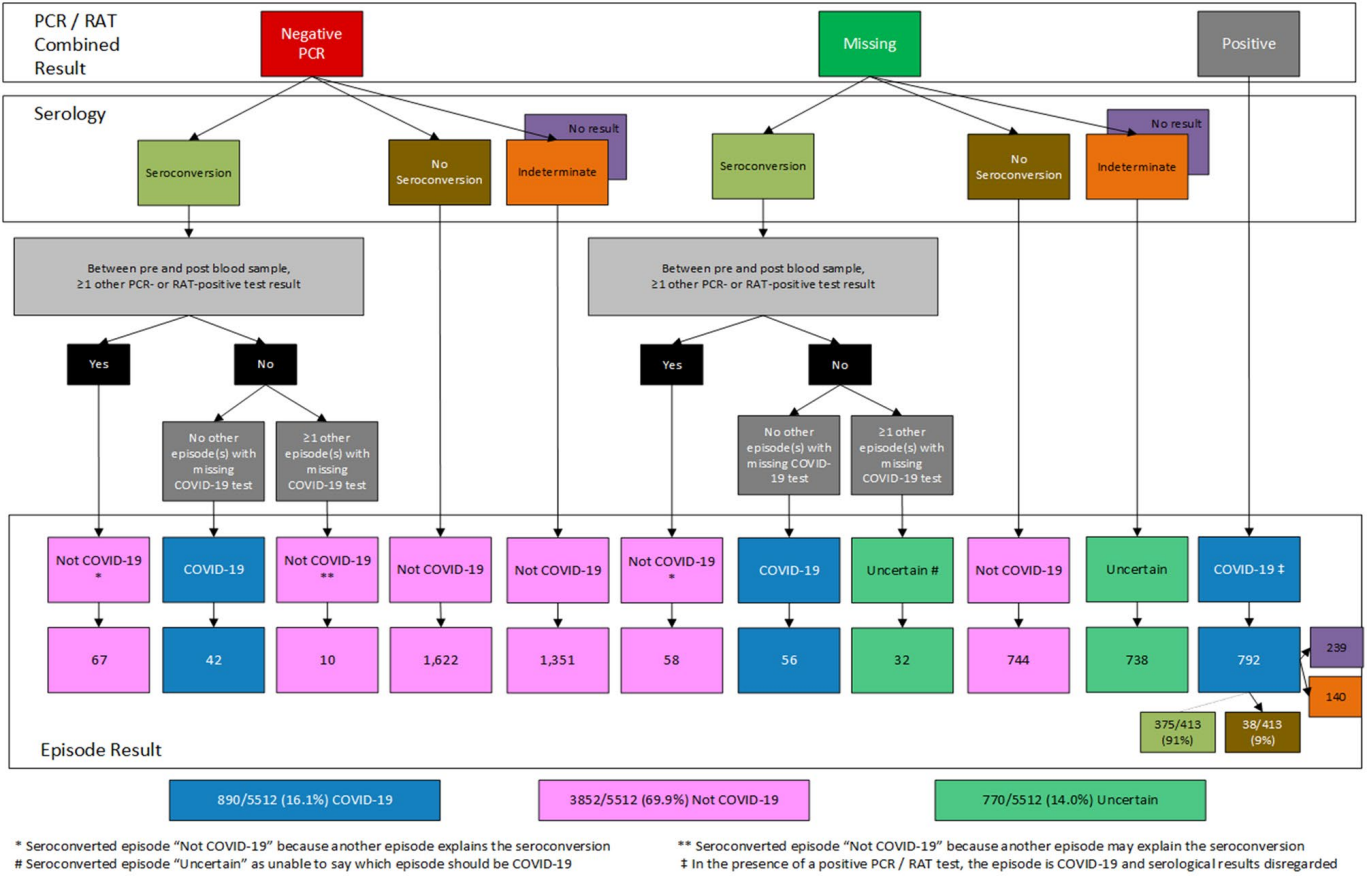
Episode interpretation algorithm: the detail

The episode interpretation algorithm produced three possible classifications for each illness episode: COVID-19, Not COVID-19 or Uncertain (which included episodes for which results were missing, or indeterminate). The results of the serological and PCR/RAT algorithms were combined to categorise illness episodes using the episode interpretation algorithm, detailed in Fig. 1. Additional conditions were then applied which evaluated other COVID-19 tests and episode results within the same timeframe.

## Results

Of the 3988 participants, 2559 reported at least one episode of illness in the year post-randomisation, with a total of 5512 episodes.

**Fig. 2:**
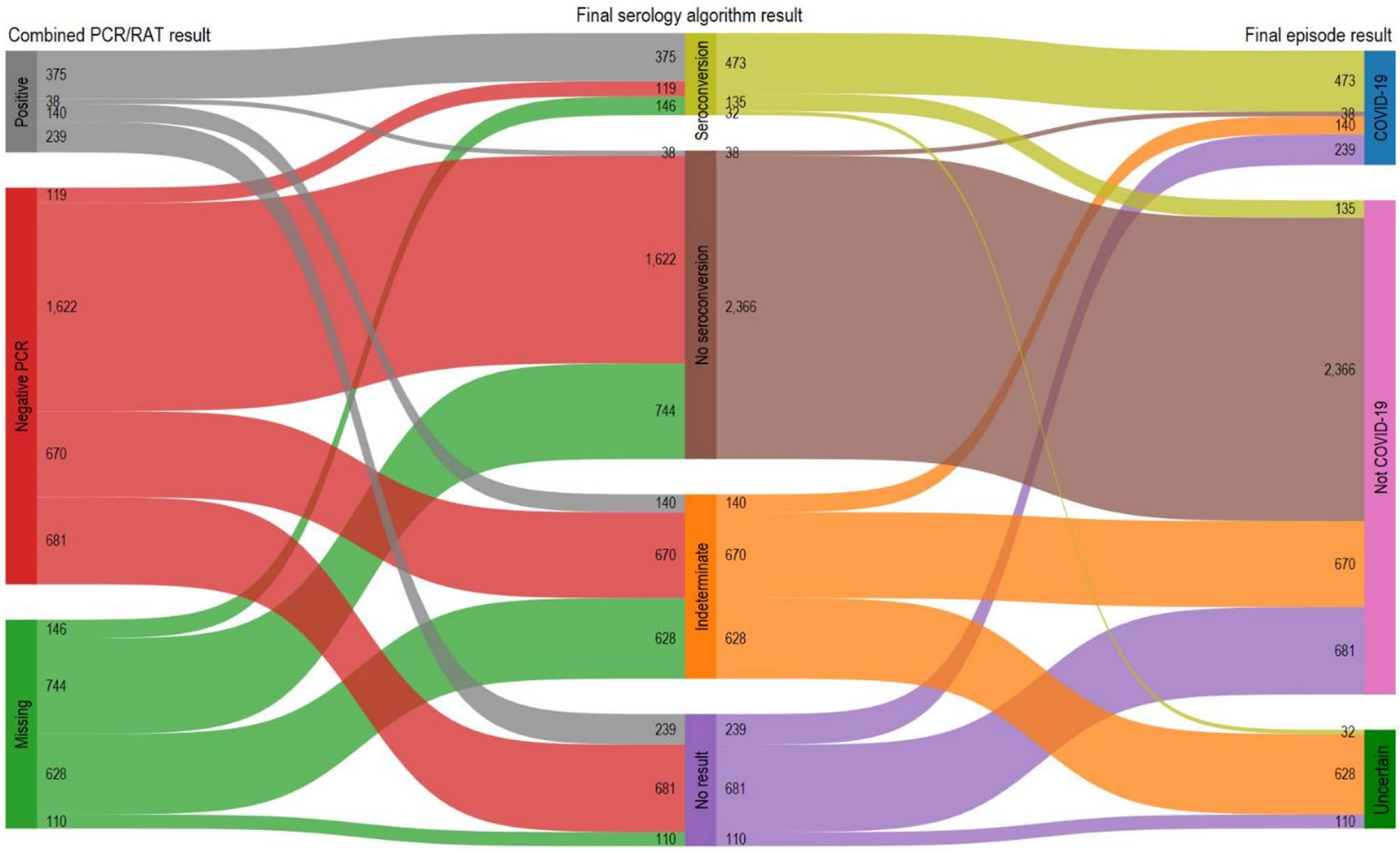
Episode interpretation algorithm

Of the 5512 episodes, 890 (16%) were classified as COVID-19, 3852 (70%) as Not COVID-19 and 770 (14%) as Uncertain (Fig. 1). The participant flow from the combined PCR/RAT result and from the pre-episode NCP serology to the final episode classification are shown in Fig. 2 and Supplementary Fig. 3 respectively.

**Fig. 3:**
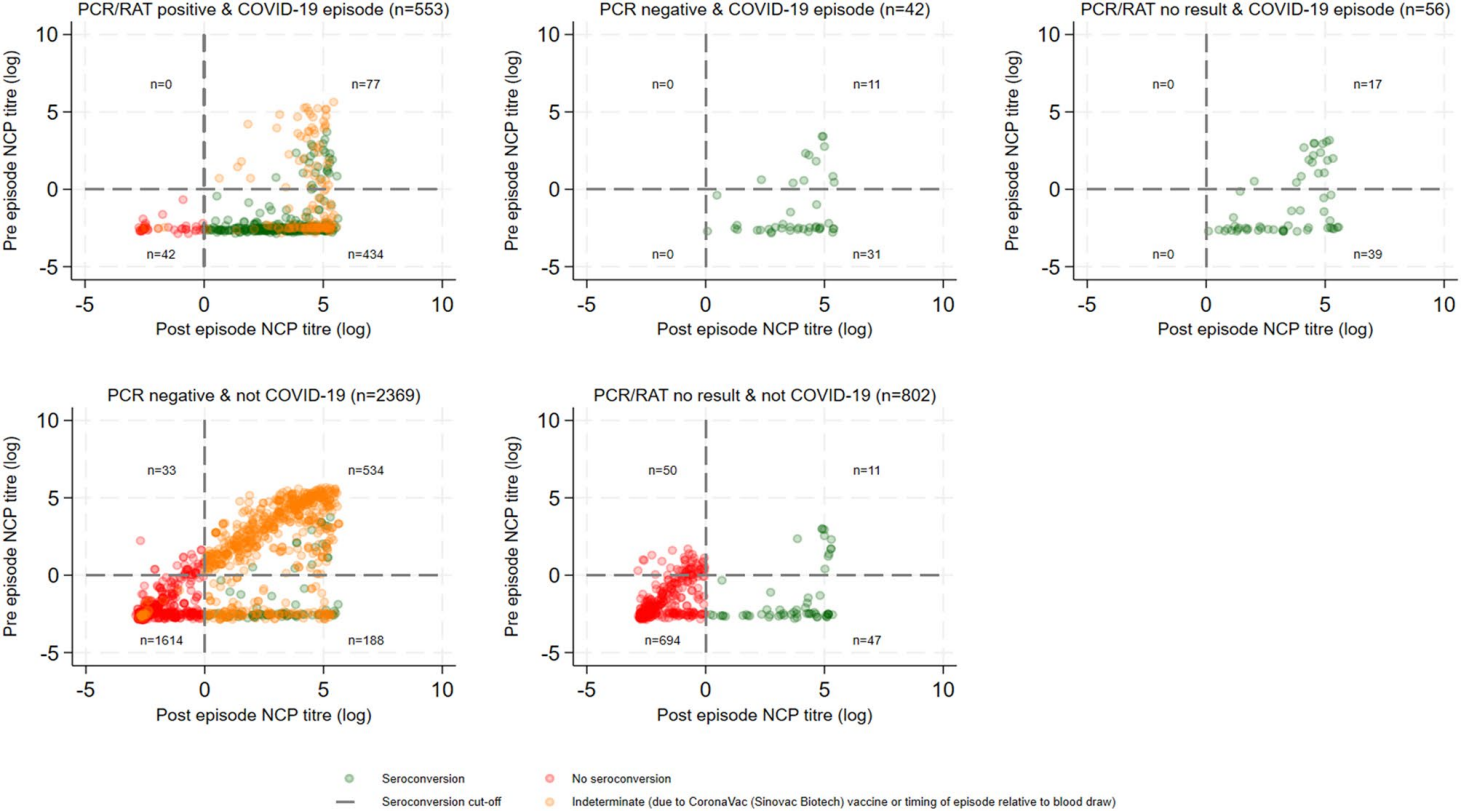
Pre and post NCP titre serology results by PCR/RAT and episode result

Of the 890 episodes defined as COVID-19, 792 (89%) had a positive PCR or RAT test result. Serology results were concordant (seroconversion) in 375 (47.4%) episodes, discordant (no seroconversion) in 38 (4.8%), and Indeterminate or No result in 379 (47.9%) (Fig. 2). The remaining 98 COVID-19 episodes comprised 56 (6.3%) with a missing PCR/RAT result and 42 (4.7%) with a negative PCR result (Supplementary Fig. 4). The discordant PCR/RAT and serology results were not attributable to post-episode NCP titres being just above the threshold for seroconversion (Fig. 3). The post-episode NCP titres in relation to the time interval between the episode start and post-episode blood collection for the seroconverted PCR negative and PCR/RAT positive episodes are shown in Supplementary Fig. 5. Median post-episode NCP titres were not significantly higher in seroconverting episodes with positive PCR/RAT results compared to those with negative PCR results (41.3, IQR: 14.3–101.4vs. median 62.2, IQR: 15.9–122.9; *p* = 0.3), episodes with positive serology and negative PCR were not merely due to NCP titres bordering the assay positivity cut-off. Relevant to this, median time between episode onset and post-episode serological testing was higher in the negative PCR episode group compared to the positive PCR/RAT group (median 76.5 days, IQR: 33–102 days vs. 61 days, IQR: 37–82 days; *p* = 0.1).

There were 305 episodes that had a negative RAT test. Of the RAT-negative episodes without a PCR result, 11/179 (6.2%) were categorised as COVID-19 based on seroconversion (Supplementary Fig. 6). In comparison, of the episodes without a PCR result, 56/858 (7.0%) were categorised as COVID-19 based on seroconversion (Fig. 3).

Among the 3852 episodes classified as Not COVID-19 by the final episode algorithm, 3050 (77%) had a negative PCR result (Fig. 2 & Supplementary Fig. 3). Of these, 1622 (53.2%) had concordant negative serology and PCR results (Fig. 2). Additionally, 1351 (44.3%) episodes had a missing NCP serology result, but a negative PCR result. The remaining 77 (2.5%) episodes were discordant (seroconversion with a negative PCR result). However, the positive NCP serology result in these cases was attributed to participants’ other PCR- or RAT-positive episodes within the same timeframe. For the remaining 802 (20%) Not COVID-19 episodes with a missing PCR result, 744 had no seroconversion between pre- and post-episode blood samples. These episodes would have been classified as Uncertain if the trial had relied on PCR/RAT results only. In the remaining 58 (7.2%) episodes, participants seroconverted, but the episode algorithm attributed this to another PCR- or RAT-positive episode.

Of the 770 episodes classified as Uncertain in the final episode result, 738 (95.8%) had both missing or indeterminant serology and PCR/RAT results. Of the remaining 32 episodes, participants seroconverted but the episodes were categorised as Uncertain because the participants had multiple episodes with missing PCR/RAT results within the same seroconversion timeframe. This precluded the attribution of the COVID-19 diagnosis to one of the episodes.

**Fig. 4:**
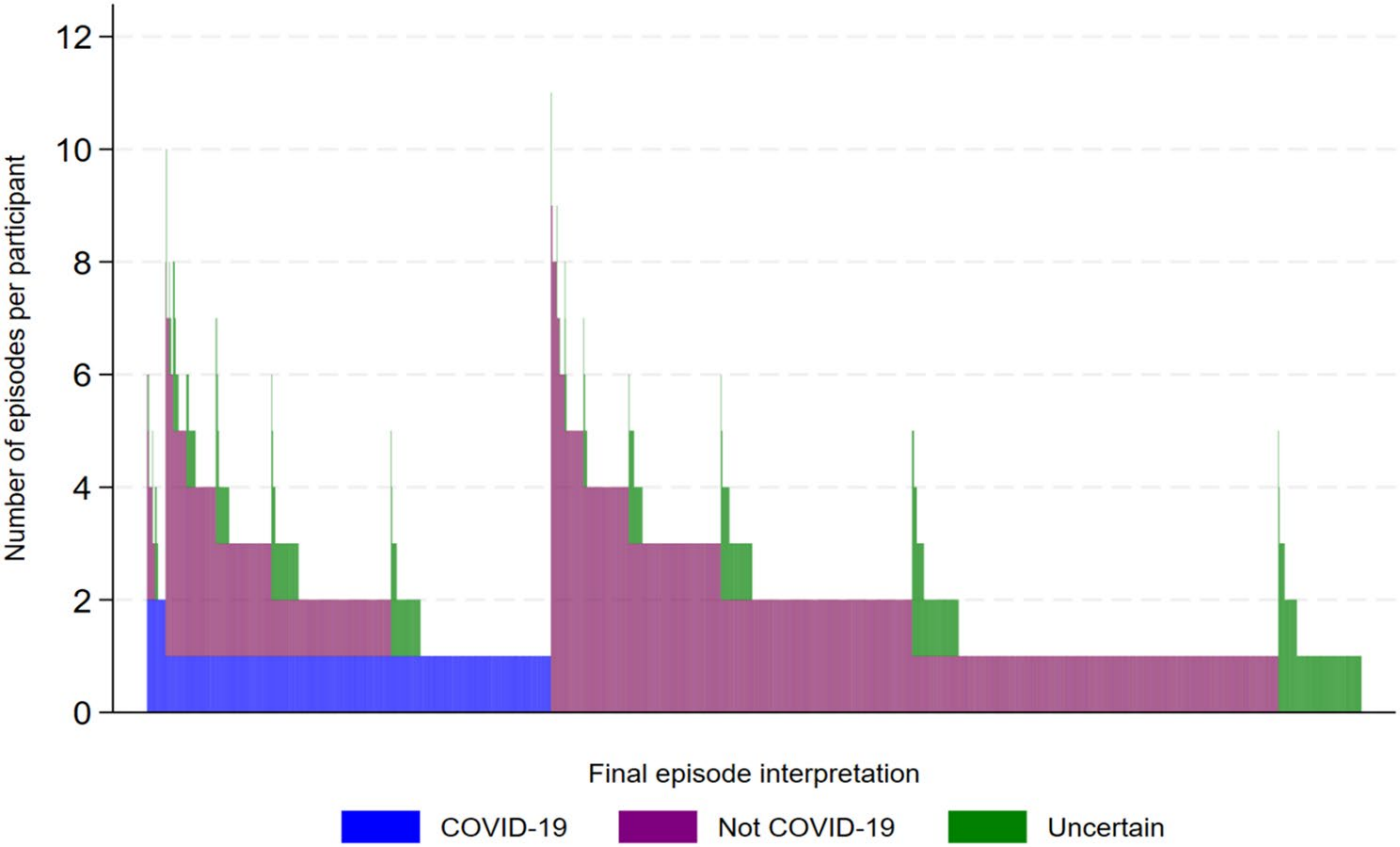
Final episode interpretation

**Fig. 5:**
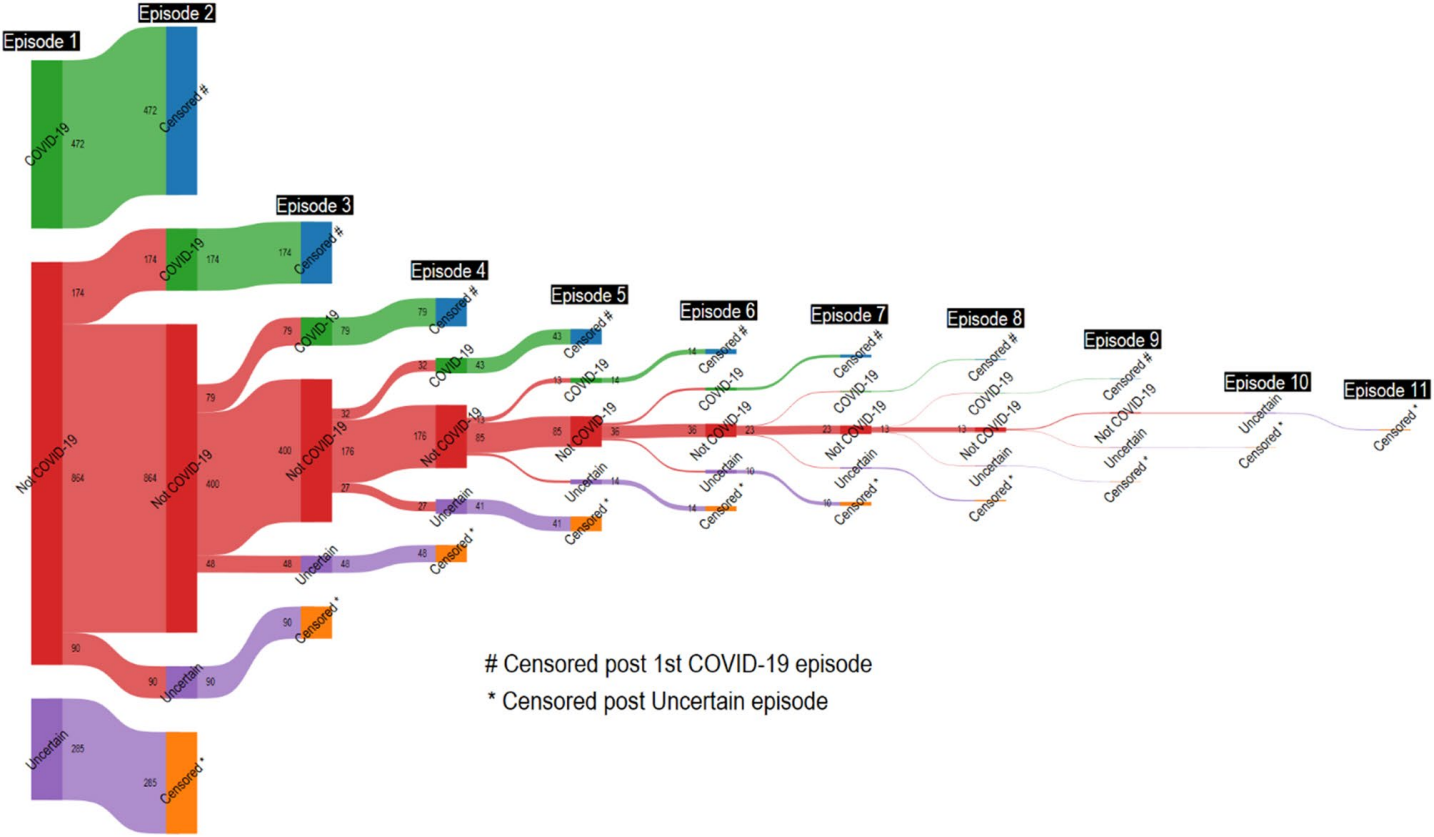
Sequential episode interpretation: COVID-19, Not COVID-19 and Uncertain outcome episodes per participant

At the participant level, Uncertain result episodes were similarly distributed between every combination of COVID-19 and Not COVID-19 episodes (Fig. 4). The sequential distribution of COVID-19, Not COVID-19 and Uncertain result episodes for each participant demonstrates the progression from algorithm processing to the final trial outcomes. It highlights the points at which participants were censored, which occurred at one of two events: their first COVID-19 episode or their first episode with an Uncertain result (Fig. 5).

## Discussion

Establishing accurate case definitions for trials with infection-related outcomes for novel pathogens, such as during the COVID‑19 pandemic, is challenging for a number of reasons. Symptom profiles often overlap with those of other respiratory illnesses; diagnostic tests evolve, and their performance might be influenced by factors such as the timing of testing and viral load. The algorithms developed for the BRACE trial provided a novel and robust approach for the classification of illness episodes and identification of COVID-19 cases. Unlike other trials that relied exclusively on PCR/RAT [10–12] or serological testing [13], often classifying cases individually [14, 15], our three-step algorithm combined these test results with real-world considerations. In the first six months of the pandemic, further information about several of these issues was emerging. It became clear that the timing of testing relative to symptom onset was critical to the sensitivity of serological testing [16], and that negative RT-PCR swabs were insufficient to rule out SARS-CoV-2 infection [17]. Moreover, variation in the sensitivity and specificity of PCR/RAT and serology became apparent [18, 19]. A review of clinical, molecular and serological methods of SARS-CoV-2 detection early in 2021 recommended a combination of these methods for optimal case detection [20]. Importantly we were able to account for the lower reliability of a negative RAT result due to its lower sensitivity [9, 21], potential false-negative PCR tests [22], and the impact of *CoronaVac*-induced anti-NCP antibodies [23]. 

Post-hoc analysis showed that less than 10% of participants with RAT-negative, PCR-missing episodes seroconverted and were classified as COVID-19, while nearly 15% of participants with RAT-negative, PCR-missing episodes were excluded from primary analysis due to missing serological results. Discordance between PCR/RAT and serology test results was observed in a small proportion (9%) of episodes. Possible explanations for the 38 (4.3%) PCR/RAT-positive episodes without seroconversion include mild infections that did not elicit a systemic response, early waning of immunity, limitations in the sensitivity of serology, or false positive PCR/RAT. Possible explanations for the 42 (4.7%) PCR-negative episodes with seroconversion which were classified as COVID-19 include inadequate swabbing, technical issues, participant reporting errors, false positive serology due to cross-reactivity or undetected asymptomatic infections during the interval between blood samples.

Serological testing proved especially valuable when PCR/RAT results were unavailable. Negative serology reclassified 744 episodes with missing PCR/RAT results as Not COVID-19 by demonstrating an absence of seroconversion. Additionally, serology alone identified 6.3% (56/890) of COVID-19 episodes in which the PCR/RAT result was missing. The algorithm was particularly beneficial in mitigating the impact of incomplete data by reducing the proportion of episodes for which classification would not have been possible (and therefore classified as Uncertain) by more than half, from 30% (1628/5512) to 14% (770/5512).

The addition of serology to PCR/RAT testing involves considerable cost and resources, including staff, consumables, shipment, laboratory assays and data management. However, the benefits included in-person follow-ups that encouraged trial retention, enhanced data accuracy, clarified discordances and the facilitation of additional research opportunities, such as additional immunological studies [24–29]. 

Our analyses have some limitations. First, our definition of symptomatic COVID-19 relied on the original case criteria, excluding non-febrile episodes without respiratory symptoms. Second, the timing and type of PCR/RAT testing were not standardised and varied depending on individual settings. Third, serological testing was done quarterly rather than at a consistent interval following illness episodes, which may have affected the detection of seroconversion. Finally, a proportion of illness episodes had incomplete data. However, it is among the COVID-19-related trials with the most complete data available.

Our three-component algorithm developed in the BRACE trial has potential for adaption to diverse epidemiological scenarios beyond COVID-19. Infectious diseases such as influenza or respiratory syncytial virus also require accurate longitudinal monitoring to track transmission, assess vaccine efficacy, and evaluate emerging diagnostics, interventions, and treatments [30, 31]. 

## Conclusions

Despite robust data collection efforts, COVID-19 trials faced practical challenges, particularly during the early pandemic. Testing availability and participant adherence contributed to the 14% of episodes still classified as missing. Adding serological testing demanded substantial time, financial resources, and participant effort but effectively halved the number of unclassified episodes. Our study underscores the important role of algorithms in achieving comprehensive classification, particularly in complex real-world study settings.

## Supplementary Information

Below is the link to the electronic supplementary material.

Supplementary Material 1

Supplementary Material 2

## Data Availability

The datasets used and/or analysed during the current study are available from the corresponding author on reasonable request and on completion of a signed data access agreement. Requests can be made in writing to [braceresearch@mcri.edu.au](mailto: braceresearch@mcri.edu.au).

## Abbreviations

COVID-19: Coronavirus Disease 2019
COI: Cut Off Index
DSMB: Data Safety and Monitory Board
HCW: Health Care Workers
NCP: Nucleocapsid Protein
PCR: Polymerase Chain Reaction
RAT: Rapid Antigen Test
SARS-CoV-2: Severe Acute Respiratory Syndrome Coronavirus 2
