# Supplementary material for: Optimising COVID-19 episode identification using serology and PCR/rapid antigen testing: insights from the BRACE trial
Source: BMC Infect Dis. 2026 Apr 7;26:974. doi: 10.1186/s12879-026-13128-6 (PMC13188293; doi:10.1186/s12879-026-13128-6)
Supplement: Supplementary file 2 — Supplementary Material 2 [file 12879_2026_13128_MOESM2_ESM.pdf]

BRACE Trial Consortium Group

[1]Prof Nigel Curtis, [1]Prof Andrew Davidson, [1]Kaya Gardiner, [1]A/Prof Amanda Gwee, [1]Tenaya Jamieson, [1]Dr Nicole Messina, [1]Thilanka Morawakage, [1]Dr Susan Perlen, [1]A/Prof Kirsten Perrett, [1]Dr Laure Pittet, [1]Amber Sastry, [1]Jia Wei Teo, [1]Francesca Orsini, [1]Prof Katherine Lee, [1]Dr Cecilia Moore, [1]Suzanna Vidmar, [1]Dr Laure Pittet, [1]Rashida Ali, [1]Ross Dunn, [1]Peta Edler, [1]Grace Gell, [1]Casey Goodall, [1]Richard Hall, [1]Ann Krastev, [1]DrNathan La, [1]Dr Ellie McDonald, [1]Nick McPhate, [1]Thao Nguyen, [1]Jack Ren, [1]Luke Stevens, [1]Dr Nicole Messina, [1]Ahmed Alamrousi, [1]Rhian Bonnici, [1]Dr Thanh Dang, [1]Susie Germano, [1]Jenny Hua, [1]Rebecca McElroy, [1]Monica Razmovska, [1]Scott Reddiex, [1]Xiaofang Wang, [1]Jeremy Anderson, [1]Kristy Azzopardi, [1]Vicki Bennett- Wood, [1]Anna Czajko, [1]Nadia Mazarakis, [1]Conor McCafferty, [1]Frances Oppedisano, [1]Belinda Ortika, [1]Casey Pell, [1]Leena Spry, [1]Ryan Toh, [1]Sunitha Velagapudi, [1]Amanda Vlahos, [1]Ashleigh Wee-Hee, [1]Pedro Ramos, [1]Karina De La Cruz, [1]Dinusha Gamage, [1]Anushka Karunanayake, [1]Isabella Mezzetti, [1]Dr Benjamin Ong, [1]Ronita Singh, [1]Enoshini Sooriyarachchi, [4]Dr Suellen Nicholson, [4]Natalie Cain, [4]Rianne Brizuela, [4]Han Huang, [1]Veronica Abruzzo, [2]Morgan Bealing, [1]Patricia Bimboese, [1]Kirsty Bowes, [1]Emma Burrell, [1]Dr Joyce Chan, [1]Jac Cushnahan, [1]Hannah Elborough, [2]Olivia Elkington, [2]Kieran Fahey, [2]Monique Fernandez, [2]Catherine Flynn, [1]Sarah Fowler, [1]Marie Gentile Andrit, [2]Bojana Gladanac, [1]Catherine Hammond, [2]Norine Ma, [2]Sam Macalister, [1]Emmah Milojevic, [2]Jesutofunmi Mojeed, [1]Jill Nguyen, [1]Liz O'Donnell, [2]Nadia Olivier, [2]Isabelle Ooi, [1]Stephanie Reynolds, [2]Lisa Shen, [1]Barb Sherry, [1]Judith Spotswood, [1]Jamie Wedderburn, [2]Angela Younes, [3]Donna Legge, [3]Jason Bell, [3]Jo Cheah, [3]Annie Cobbledick, [3]Kee Lim, [3]Sonja Elia, [3]Lynne Addlem, [1]Anna Bourke, [1]Clare Brophy, [3]Nadine Henare, [3]Narelle Jenkins, [1]Francesca Machingaifa, [3]Skye Miller, [1]Kirsten Mitchell, [3]Sigrid Pitkin, [3]Kate Wall, [1]Dr Paola Villanueva, [1]A/Prof Nigel Crawford, [1]Dr Laure Pittet, [1]Dr Wendy Norton, [5]Dr Niki Tan, [5]Thilakavathi Chengodu, [5]Diane Dawson, [5]Victoria Gordon, [6]Tony Korman, [6]Jess O'Bryan, [1]Sophie Agius, [1]Dr Samantha Bannister, [1]Jess Bucholc, [1]Alison Burns, [1]Beatriz Camesella, [1]Prof John Carlin, [1]Marianna Ciaverella, [1]Maxwell Curtis, [1]Stephanie Firth, [1]Dr Christina Guo, [1]Matthew Hannan, [1]Erin Hill, [1]Sri Joshi, [1]Katherine Lieschke, [1]Megan Mathers, [1]Sasha Odoi, [1]Ashleigh Rak, [1]Dr Chris Richards, [1]Leah Steve, [1]Carolyn Stewart, [1]Dr Eva Sudbury, [1]Helen Thomson, [1]Emma Watts, [1]Fiona Williams, [1]Angela Young, [1]Penny Glenn, [1]Andrew Kaynes, [1]Amandine Philippart De Floy, [7]Sandy Buchanan, [1]Thijs Sondag, [8]Ivy Xie, [1]Harriet Edmund, [1]Bridie Byrne, [1]Tom Keeble, [1]Belle Ngien, [1]Fran Noonan, [1]Michelle Wearing-Smith, [9]Alison Clarke, [9]Pemma Davies, [9]Oliver Eastwood, [9]Alric Ellinghaus, [9]Rachid Ghieh, [9]Zahra Hilton, [9]Emma Jennings, [9]Athina Kakkos, [9]Iris Liang, [9]Katie Nicol, [9]Sally O'Callaghan, [9]Helen Osman, [9]Gowri Rajaram, [9]Sophia Ratcliffe, [9]Victoria Rayner, [9]Ashleigh Salmon, [9]Angela Scheppokat, [9]Aimee Stevens, [9]Rebekah Street, [9]Nicholas Toogood, [10]A/Prof Nicholas Wood, [10]Twinkle Bahaduri, [10]Therese Baulman, [10]Jennifer Byrne, [10]Candace Carter, [10]Mary Corbett, [10]Aiken Dao, [10]Maria Desylva, [10]Dr Andrew Dunn, [10]Evangeline Gardiner, [10]Rosemary Joyce, [10]Dr Rama Kandasamy, [10]Prof Craig Munns, [10]Lisa Pelayo, [10]Dr Ketaki Sharma, [10]Katrina Sterling, [10]Caitlin Uren, [11]Clinton Colaco, [11]A/Prof Mark Douglas, [11]Kate Hamilton, [12]Dr Adam Bartlett, [12]Dr Brendan McMullan, [12]Dr Pamela Palasanthiran, [12]Dr Phoebe Williams, [13]Dr Justin Beardsley, [13]Nikki Bergant, [13]Renier Lagunday, [13]Dr Kristen Overton, [13]Prof Jeffrey Post, [14]Dr Yasmeen Al- Hindawi, [14]Sarah Barney, [14]A/Prof Anthony Byrne, [14]Lee Mead, [14]Marshall Plit, [15]Prof. David Lynn, [15]Saoirse Benson, [15]Dr Stephen Blake, [15]Rochelle Botten, [15]Tee Yee Chern, [15]Georgina Eden, [15]Liddy Griffith, [15]Jane James, [15]Dr Miriam Lynn, [15]Angela Markow, [15]Domenic Sacca, [15]Dr Natalie Stevens, [15]Prof. Steve Wesselingh, [16]Catriona Doran, [16]Dr Simone Barry, [16]Dr Alice Sawka, [17]Dr Sue Evans, [17]Louise Goodchild, [17]Christine Heath, [17]Meredith Krieg, [17]Prof. Helen Marshall, [17]Mark McMillan, [17]Mary Walker, [18]Prof Peter Richmond, [18]Nelly Amenyogbe, [18]Christina Anthony, [18]Annabelle Arnold, [18]Beth Arrowsmith, [18]Rym Ben-Othman, [18]Sharon Clark, [18]Jemma Dunnill, [18]Nat Eiffler, [18]Krist Ewe, [18]Carolyn Finucane, [18]Lorraine Flynn, [18]Camille Gibson, [18]Lucy Hartnell, [18]Elysia Hollams, [18]Heidi Hutton, [18]Lance Jarvis, [18]Jane Jones, [18]Jan Jones, [18]Karen

Jones, [18]Jennifer Kent, [18]Prof Tobias Kollmann, [18]Debbie Lalich, [18]Wenna Lee, [18]Rachel Lim, [18]Sonia McAlister, [18]Fiona McDonald, [18]Andrea Meehan, [18]Asma Minhaj, [18]Lisa Montgomery, [18]Melissa O'Donnell, [18]Jaslyn Ong, [18]Joanne Ong, [18]Kimberley Parkin, [18]Glady Perez, [18]Catherine Power, [18]Shadie Rezazadeh, [18]Holly Richmond, [18]Sally Rogers, [18]Nikki Schultz, [18]Margaret Shave, [18]Patrycja Skut, [18]Lisa Stiglmayer, [18]Alexandra Truelove, [18]Dr Ushma Wadia, [18]Rachael Wallace, [18]Justin Waring, [19]Michelle England, [19]Erin Latkovic, [19]A/Prof Laurens Manning, [20]Dr Susan Herrmann, [20]Prof Michaela Lucas, [21]Dr Marcus Lacerda, [21]Paulo Henrique Andrade, [21]Fabiane Bianca Barbosa, [21]Dayanne Barros, [21]Larissa Brasil, [21]Ana Greyce Capella, [21]Ramon Castro, [21]Erlane Costa, [21]Dilcimar de Souza, [21]Maianne Dias, [21]José Dias, [21]Klenilson Ferreira, [21]Paula Figueiredo, [21]Thamires Freitas, [21]Ana Carolina Furtado, [21]Larissa Gama, [21]Vanessa Godinho, [21]Cintia Gouy, [21]Daniele Hinojosa, [21]Dr Bruno Jardim, [21]Dr Tyane Jardim, [21]Joel Junior, [21]Augustto Lima, [21]Bernardo Maia, [21]Adriana Marins, [21]Kelry Mazurega, [21]Tercilene Medeiros, [21]Rosangela Melo, [21]Marinete Moraes, [21]Elizandra Nascimento, [21]Juliana Neves, [21]Maria Gabriela Oliveira, [21]Thais Oliveira, [21]Ingrid Oliveira, [21]Arthur Otsuka, [21]Rayssa Paes, [21]Handerson Pereira, [21]Gabrielle Pereira, [21]Christiane Prado, [21]Evelyn Queiroz, [21]Laleyska Rodrigues, [21]Bebeto Rodrigues, [21]Dr Vanderson Sampaio, [21]Anna Gabriela Santos, [21]Daniel Santos, [21]Tilza Santos, [21]Evelyn Santos, [21]Ariandra Sartim, [21]Ana Beatriz Silva, [21]Juliana Silva, [21]Emanuelle Silva, [21]Mariana Simão, [21]Caroline Soares, [21]Antonny Sousa, [21]Alexandre Trindade, [21]Dr Fernando Val, [21]Adria Vasconcelos, [21]Heline Vasconcelos, [22]Prof Julio Croda, [22]Carolinne Abreu, [22]Katya Martinez Almeida, [22]Camila Bitencourt de Andrade, [22]Jhenyfer Thalyta Campos Angelo, [22]Ghislaine Gonçalvez de Araújo Arcanjo, [22]Bianca Maria Silva Menezes Arruda, [22]Wellyngthon Espindola Ayala, [22]Adelita Agripina Refosco Barbosa, [22]Felipe Zampieri Vieira Batista, [22]Fabiani de Morais Batista, [22]Miriam de Jesus Costa, [22]Dr Mariana Garcia Croda, [22]Lais Alves da Cruz, [22]Roberta Carolina Pereira Diogo, [22]Rodrigo Cezar Dutra Escobar, [22]Iara Rodrigues Fernandes, [22]Leticia Ramires Figueiredo, [22]Leandro Galdino Cavalcanti Gonçalves, [22]Sarita Lahdo, [22]Joyce dos Santos Lencina, [22]Guilherme Teodoro de Lima, [22]Larissa Santos Matos, [22]Bruna Tayara Leopoldina Meireles, [22]Debora Quadros Moreira, [22]Lilian Batista Silva Muranaka, [22]Adriely de Oliveira, [22]Karla Regina Warszawski de Oliveira, [22]Matheus Vieira de Oliveira, [22]Prof Roberto Dias de Oliveira, [22]Andrea Antonia Souza de Almeida dos Reis Pereira, [22]Marco Puga, [22]Caroliny Veron Ramos, [22]Thaynara Haynara Souza da Rosa, [22]Karla Lopes dos Santos, [22]Claudinalva Ribeiro dos Santos, [22]Dyenyffer Stéffany Leopoldina dos Santos, [22]Karina Marques Santos, [22]Paulo César Pereira da Silva, [22]Paulo Victor Rocha da Silva, [22]Débora dos Santos Silva, [22]Patricia Vieira da Silva, [22]Bruno Freitas da Rosa Soares, [22]Mariana Gazzoni Sperotto, [22]Mariana Mayumi Tadokoro, [22]Daniel Tsuha, [22]Hugo Miguel Ramos Vieira, [23]Prof Margareth Maria Pretti Dalcolmo, [23]Cíntia Maria Lopes Alves da Paixão, [23]Gabriela Corrêa E Castro, [23]Simone Silva Collopy, [23]Renato da Costa Silva, [23]Samyra Almeida da Silveira, [23]Alda Maria Da-Cruz, [23]Alessandra Maria da Silva Passos de Carvalho, [23]Rita de Cássia Batista, [23]Maria Luciana Silva De Freitas, [23]Aline Gerhardt de Oliveira Ferreira, [23]Ana Paula Conceição de Souza, [23]Paola Cerbino Doblas, [23]Ayla Alcoforado da Silva dos Santos, [23]Vanessa Cristine de Moraes dos Santos, [23]Dayane Alves dos Santos Gomes, [23]Anderson Lage Fortunato, [23]Adriano Gomes-Silva, [23]Monique Pinto Gonçalves, [23]Paulo Leandro Garcia Meireles Junior, [23]Estela Martins da Costa Carvalho, [23]Fernando do Couto Motta, [23]Ligia Maria Olivo de Mendonça, [23]Girlene dos Santos Pandine, [23]Rosa Maria Plácido Pereira, [23]Ivan Ramos Maia, [23]Jorge Luiz da Rocha, [23]João Victor Paiva Romano, [23]Glauce dos Santos, [23]Erica Fernandes da Silva, [23]Marilda Agudo Mendonça Teixeira de Siqueira, [23]Ágatha Cristinne Prudêncio Soares, [24]Prof Marc Bonten, [24]Sandra Franch Arroyo, [24]Henny Ophorst-den Besten, [24]Anna Boon, [24]Karin M Brakke, [24]Axel Janssen, [24]Marijke A.H. Koopmans, [24]Toos Lemmens, [24]Titia Leurink, [24]A/Prof Cristina Prat-Aymerich, [24]Engelien Septer-Bijleveld, [24]Kimberly Stadhouders, [24]Dr Darren Troeman, [24]Marije van der Waal, [24]Marjoleine van Opdorp, [24]Nicolette van Sluis, [24]Beatrijs Wolters, [25]Prof Jan Kluytmans, [25]Jannie Romme, [25]Dr Wouter van den Bijllaardt, [25]Linda van Mook, [25]Dr M.M.L (Miranda) van Rijen, [25]P. M. G. Filius, [25]Jet Gisolf, [25]Frances Greven, [25]Danique Huijbens, [25]Dr Robert Jan Hassing, [25]R. C. Pon, [25]Lieke

Preijers, [25]J. H. van Leusen, [25]Harald Verheij, [26]Dr Wim Boersma, [26]Evelien Brans, [26]Paul Kloeg, [26]Kitty Molenaar-Groot, [26]Nhat Khanh Nguyen, [26]Dr Nienke Paternotte, [26]Anke Rol, [26]Lida Stooper, [27]Helga Dijkstra, [27]Esther Eggenhuizen, [27]Lucas Huijs, [27]Dr Simone Moorlag, [27]Prof Mihai Netea, [27]Eva Pranger, [27]Dr Esther Taks, [27]Dr Jaap ten Oever, [27]Rob ter Heine, [28]Kitty Blauwendraat, [28]Dr Bob Meek, [28]Isil Erkaya, [28]Houda Harbech, [28]Dr Nienke Roescher, [28]Rifka Peeters, [28]Menno te Riele, [28]Carmen Zhou, [29]Dr Esther Calbo, [29]Cristina Badia Marti, [29]Emma Triviño Palomares, [29]Tomás Perez Porcuna, [30]Anabel Barriocanal, [30]Ana Maria Barriocanal, [30]Irma Casas, [30]Jose Dominguez, [30]Maria Esteve, [30]Alicia Lacoma, [30]Irene Latorre, [30]Gemma Molina, [30]Barbara Molina, [30]Dr Antoni Rosell, [30]Sandra Vidal, [31]Lydia Barrera, [31]Natalia Bustos, [31]Ines Portillo Calderón, [31]David Gutierrez Campos, [31]Jose Manuel Carretero, [31]Angel Dominguez Castellano, [31]Renato Compagnone, [31]Encarnacion Ramirez de Arellano, [31]Almudena de la Serna, [31]Maria Dolores del Toro Lopez, [31]Marie-Alix Clement Espindola, [31]Ana Belen Martin Gutierrez, [31]Alvaro Pascual Hernandez, [31]Virginia Palomo Jiménez, [31]Elisa Moreno, [31]Nicolas Navarrete, [31]Teresa Rodriguez Paño, [31]Prof Jesús Rodríguez-Baño, [31]Enriqueta Tristán, [31]Maria Jose Rios Villegas, [32]Atsegiñe Canga Garces, [32]Erika Castro Amo, [32]Raquel Coya Guerrero, [32]Dr. Josune Goikoetxea, [32]Leticia Jorge, [32]Cristina Perez, [33]Dr María Carmen Fariñas Álvarez, [33]Manuel Gutierrez Cuadra, [33]Dr Francisco Arnaiz de las Revillas Almajano, [33]Pilar Bohedo Garcia, [33]Dr Teresa Giménez Poderos, [33]Claudia González Rico, [33]Blanca Sanchez, [33]Olga Valero, [33]Noelia Vega, [34]Prof John Campbell, [34]Anna Barnes, [34]Dr Helen Catterick, [34]Tim Cranston, [34]Phoebe Dawe, [34]Emily Fletcher, [34]Liam Fouracre, [34]Dr Alison Gifford, [34]Prof Neil Gow, [34]John Kirkwood, [34]Dr Christopher Martin, [34]Dr Amy McAndrew, [34]Marcus Mitchell, [34]Georgina Newman, [34]Dr Abby O'Connell, [34]Jakob Onysk, [34]Lynne Quinn, [34]Dr Shelley Rhodes, [34]Samuel Stone, [34]Dr Lorrie Symons, [34]Harry Tripp, [34]Prof Adilia Warris, [34]Darcy Watkins, [34]Bethany Whale, [35]Dr Alex Harding, [35]Gemma Lockhart, [35]Dr Kate Sidaway-Lee, [36]Dr John Campbell, [36]Dr Sam Hilton, [36]Sarah Manton, [36]Dr Daniel Webber-Rookes, [36]Rachel Winder, [37]James Moore, [38]Freya Bateman, [38]Dr Michael Gibbons, [38]Dr Bridget Knight, [38]Julie Moss, [38]Dr Sarah Statton, [38]Josephine Studham, [39]Lydia Hall, [39]Will Moyle, [39]Dr Tamsin Venton

[1] Murdoch Children's Research Insitute, Parkville, Victoria, Australia
[2] The University of Melbourne, Melbourne, Victoria, Australia
[3] The Royal Children's Hospital Melbourne, Parkville, Victoria, Australi
[4] Victorian Infectious Disease Reference Laboratory, Melbourne, Victoria, Australia
[5] Epworth, Richmond, Victoria, Australia
[6] Monash Health, Clayton, Victoria, Australia
[7] WeGuide, Parkville, Victoria, Australia
[8] Curve Tomorrow, Parkville, Victoria, Australia
[9] Orygen, Parkville, Victoria, Australia
[10] The Children's Hospital At Westmead, Westmead, NSW, Australia
[11] Westmead Hospital, Westmead, NSW, Australia
[12] Sydney Children's Hospital, Randwick, NSW, Australia
[13] Prince of Wales Hospital, Randwick, NSW, Asutralia
[14] St Vincent's Hopsital Sydney, Darlinghurst, NSW, Australia
[15] South Australian Health and Medical Research Institute, Adelaide, SA, Australia
[16] Royal Adelaide Hospital, Adelaide, SA, Australia
[17] Women's and Children's Hospital, North Adelaide, SA, Australia
[18] Perth Children's Hopsital/The Kids Institute Australia, Nedlands, WA, Australia
[19] Fiona Stanley Hospital, Murdoch, WA, Australia
[20] Sir Charles Gairdner Hospital, Nedlands, WA, Australia
[21] Doctor Heitor Vieira Dourado Tropical Medicine Foundation, Manaus, Amazonas, Brazil

[22] Fundação Oswaldo Cruz, Campo Grande,Mato Grosso do Sul, Brazil Mato Grosso do Sul
[23] Centro de Referência Professor Hélio Fraga, ENSP/FIOCRUZ (Fundação Oswaldo Cruz), Rio de Janeiro, Brazil.
[24] UMC Utrect, Utrect, Netherlands
[25] Amphia Hospital, Breda, Netherlands
[26] Noord West Ziekenhuis, Den Helder, Netherlands
[27] Radboud University Medical Center, Nijmegen, Netherlands
[28] St Antonius Hospital, Nieuwegein, Netherlands
[29] Mutua Terrassa University Hospital, Barcelona, Spain
[30] University Hospital Germans Trias I Pujol, Badalona, Barcelona, Spain
[31] Hospital Virgen Macarena, Sevilla, Spain
[32] University Hospital Cruces, Barakaldo, Bizkaia, Spain
[33] Marqués de Valdecilla University Hospital, Santander, Cantabria, Spain
[34] University of Exeter/Exeter Clinical Trials Unit, Exeter, Devon, United Kingdom
[35] St Leonard's Practice, Exeter, Devon, United Kingdom
[36] Ide Lane Surgery, Exeter, Devon, UK
[37] Travel Clinic, Exeter, Devon, UK
[38] Royal Devon and Exeter NHS Foundation Trust, Exeter, Devon, UK
[39] Teign Estuary Medical Group/Glendevon Medical Practice, Teignmouth, Devon, UK
